# Supplementary material for: Integrative network analysis interweaves the missing links in cardiomyopathy diseasome
Source: Sci Rep. 2022 Nov 16;12:19670. doi: 10.1038/s41598-022-24246-x (PMC9668833; doi:10.1038/s41598-022-24246-x)
Supplement: Supplementary file 11 — Supplementary Information 11. [file 41598_2022_24246_MOESM11_ESM.docx]

**Supplementary Information**

**Integrative network analysis interweaves the missing links in cardiomyopathy diseasome**

Pankaj Kumar Chauhan & Ramanathan Sowdhamini*

National Centre for Biological Sciences (Tata Institute of Fundamental Research), GKVK Campus, Bangalore Karnataka 560065 INDIA

*Correspondence: [mini@ncbs.res.in](mailto:mini@ncbs.res.in)

**Supplementary Figures and Tables**


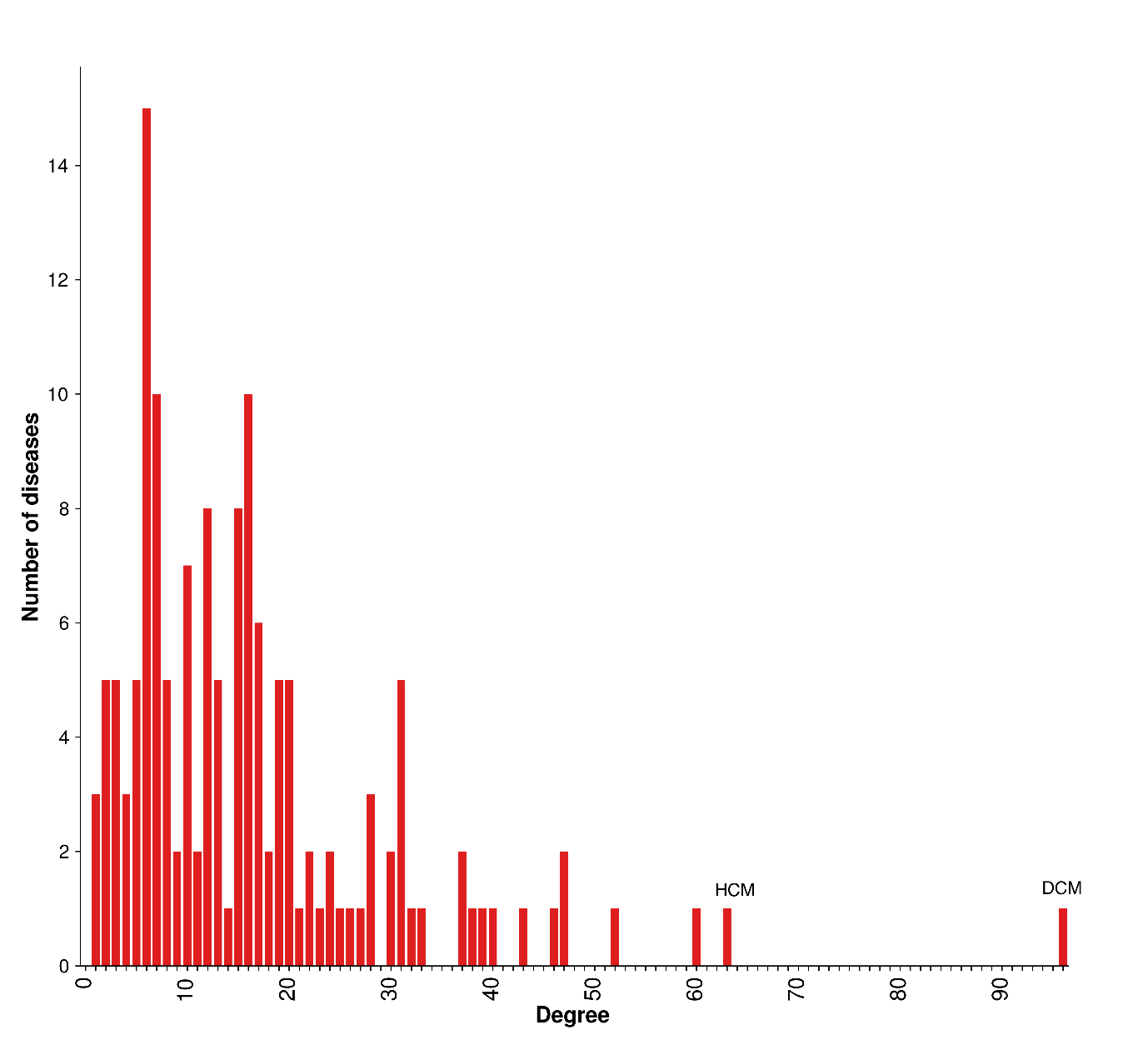


**Figure S1: Cardiomyopathy diseasome connectivity distribution, related to Figure 2.**

This figure shows connectivity distribution of cardiomyopathy diseasome. Majority of diseases are connected to a few other diseases only. DCM and HCM show high connectivity with k-value of 96 and 63 respectively.


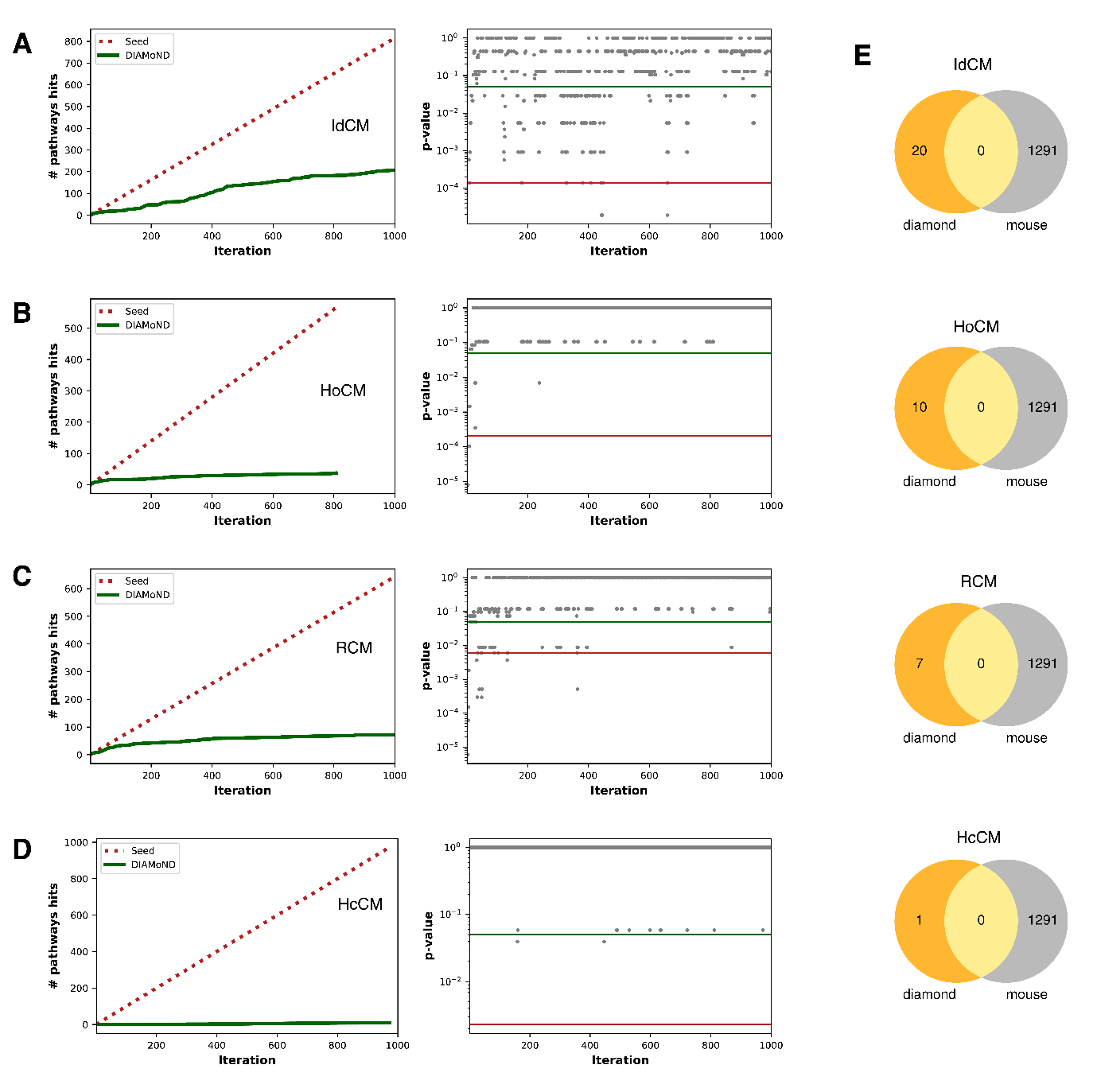


**Figure S2: Boundary estimation of the predicted** **DIAMOnD genes, related to figure 4.**

Graph illustration of the biological validation of the predicted DIAMOnD genes. (**A,B,C,D**) Panels correspond to true molecular pathways hits and corresponding p-values of DIAMOnD genes of idiopathic cardiomyopathy (**A**), hypertrophic obstructive cardiomyopathy (**B**), restrictive cardiomyopathy (**C**), and histocoid cardiomyopathy (**D**), respectively. These pathways were firstly enriched (adjusted p-value = 0.05) using seed genes of individual cardiomyopathy. In the pathways hits plot, red lines depict seed genes and green lines refer to the DIAMOnD genes. In the p-value plot, red line highlights p-value of seed genes and green line marks the p-value = 0.05. **E.** Venn diagrams showing overlap between predicted DIAMOnD genes and genes showing abnormal heart phenotype in mouse for the above diseases.


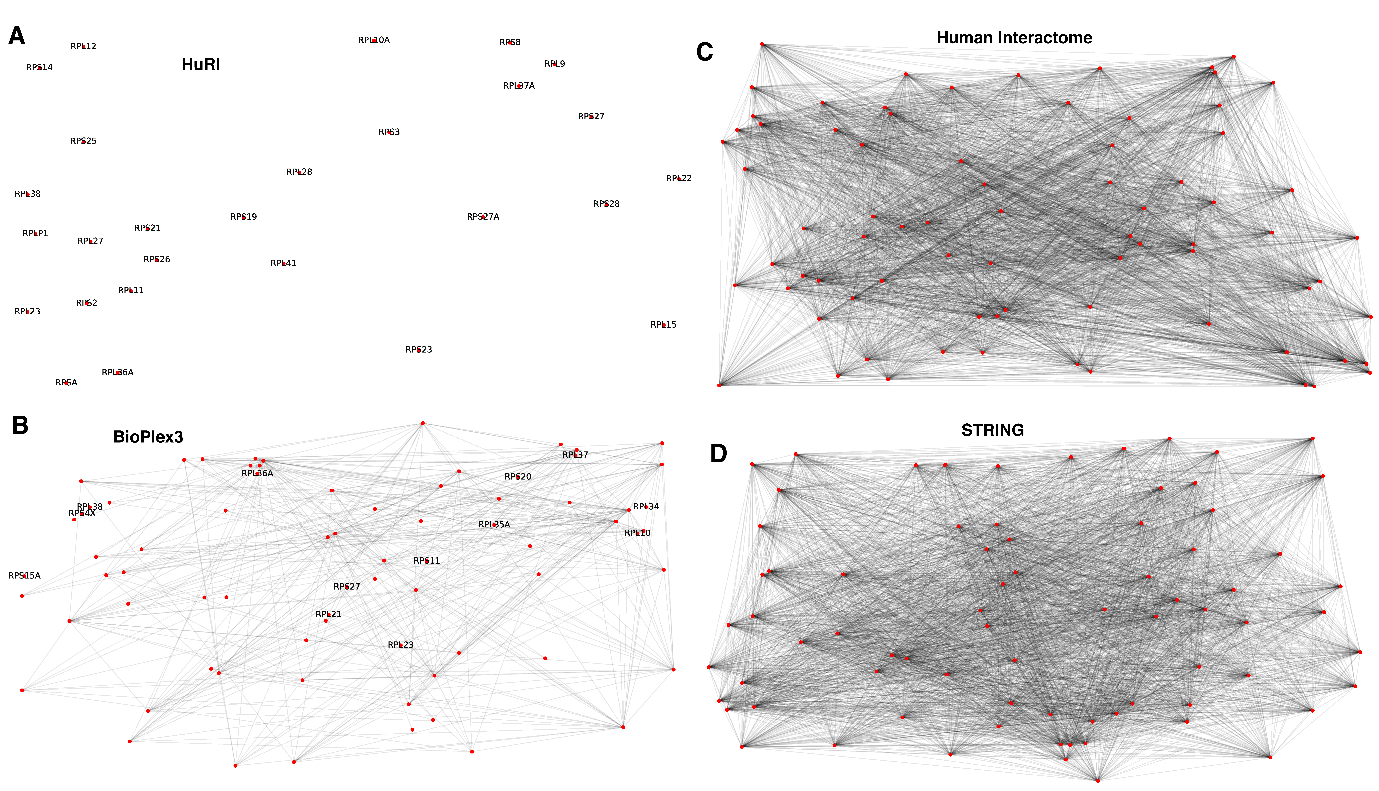


**Figure S3: Interaction coverage of the human ribosomal complex proteins in reference datasets, related to Figure 4.**

Network visualization of the ribosomal complex interactions in HuRI (**A**), Bioplex3 (**B**), our human interactome (**C**), and STRING (**D**) datasets. The isolated proteins in each dataset are labelled to distinguish them in the network.

**Table S1: Disease-gene genetic data**

List of the all diseases considered in the study.

**Table S2: Cardiomyopathies data**

Various cardiomyopathies and genes implicated in them.

**Table S3: Cardiomyopathy diseasome bipartite network**

A bipartite network data for the diseases with at least one cardiomyopathy gene.

**Table S4: Cardiomyopathy diseasome network**

The disease projection data of the bipartite network.

**Table S5: Network properties of the cardiomyopathy diseasome**

List of the Degree Centrality (DC), Betweenness Centrality (BC), Closeness Centrality (CC) and Clustering for each disease in the cardiomyopathy diseasome. These network statistics show the connectivity of the diseasome.

**Table S6: Modifier genes in cardiomyopathies**

List of the screened DIAMOnD genes in the major cardiomyopathies (HCM, DCM, ACM, IdCM, RCM, HoCM and HcCM).

**Table S7: Modifier genes with abnormal mouse heart phenotype ortholog**

List of the screened DIAMOnD genes with abnormal mouse heart phenotype ortholog in the HCM, DCM and ACM.

**Table S8: Modifier genes in disease-disease association**

List of new cardiomyopathy and other diseases association due to the modifier genes.

**Table S9: Modifier genes’ disease-disease association frequency**

List of major modifier genes in terms of frequency in the disease-disease association.

**Table S10: Modifier genes’ tissue expression**

The RNA expression (pTPM) of the modifier genes in the HPA dataset.
